# S12-3: How to empower women in difficult life situations to engage in physical activity using a human rights approach?

**DOI:** 10.1093/eurpub/ckae114.253

**Published:** 2024-09-26

**Authors:** Karim Abu-Omar, Raluca Sommer, Farah Sweidan, Maike Till, Heiko Ziemainz

**Affiliations:** FAU Erlangen, Department of Sport Science and Sport, Erlangen, Germany; FAU Erlangen, Department of Sport Science and Sport, Erlangen, Germany; FAU Erlangen, Department of Sport Science and Sport, Erlangen, Germany; FAU Erlangen, Department of Sport Science and Sport, Erlangen, Germany; FAU Erlangen, Department of Sport Science and Sport, Erlangen, Germany

## Abstract

**Purpose:**

In the realm of daily life, physical activity should constitute a fundamental component of the people’s routines. However, specific vulnerable population groups (e.g., single mothers, unemployed women, women with low educational attainment) are disadvantaged when seeking to engage in physical activity. By employing a human rights approach, it is crucial to evaluate this issue in order to ensure that disadvantaged women can genuinely exercise their right to participate in physical activity. BIG, a community-based participatory research project, aims to engage disadvantaged women in physical activity through tailored exercise courses and social activities.

**Methods:**

12 semi-structured interviews were conducted with seven women participating in various physical activity courses provided by the BIG program and five project coordinators implementing BIG in different communities. The interviews were analyzed by two researchers using deductive content analysis with a focus on the four human rights criteria.

**Results:**

Despite the presence of various local physical activity offers, women perceive a lack of suitable choices, especially considering their culture and familial circumstances (e.g., small children, Ramadan-feast). BIG aims to address this by creating new offers (availability) and improving accessibility to existing ones. Certain courses hinder women’s participation by lacking a “safe space” for them (e.g., mixed-gender classes), impacting accessibility. Culturally sensitive offers, like schedules aligned with Muslim prayer times, increase acceptability. To ensure the effective implementation of appropriate courses, intercultural competence training was integrated into relevant trainings, enhancing the overall quality of BIG.

**Conclusions:**

Using a human rights-based approach and its four key criteria allows the identification of shortcomings in the promotion of physical activity among disadvantaged women.

**Funding source:**

This research received funding from the German Federal Ministry of Education and Research (NU-BIG, 01EL2012A) and the National Association of Statutory Health Insurance Funds (BIG-5, Z2/95022001).

